# A Hierarchical Classification of Benthic Biodiversity and Assessment of Protected Areas in the Southern Ocean

**DOI:** 10.1371/journal.pone.0100551

**Published:** 2014-07-17

**Authors:** Lucinda L. Douglass, Joel Turner, Hedley S. Grantham, Stefanie Kaiser, Andrew Constable, Rob Nicoll, Ben Raymond, Alexandra Post, Angelika Brandt, Daniel Beaver

**Affiliations:** 1 Centre for Biodiversity and Conservation Science, School of Biological Sciences, The University of Queensland, Brisbane, Queensland, Australia; 2 Centre for Conservation Geography, Sydney, New South Wales, Australia; 3 Betty and Gordon Moore Centre for Science and Oceans, Conservation International, Arlington, Virginia, United States of America; 4 Biocentre Grindel and Zoological Museum, University of Hamburg, Hamburg, Germany; 5 German Centre for Marine Biodiversity Research, Wilhelmshaven, Germany; 6 Australian Antarctic Division, Department of the Environment, Australian Government, Kingston, Tasmania, Australia; 7 Antarctic Climate and Ecosystems Cooperative Research Centre, University of Tasmania, Hobart, Tasmania, Australia; 8 WWF Australia, Ultimo, New South Wales, Australia; 9 Marine and Coastal Environment Group, Geoscience Australia, Canberra, Australian Capital Territory, Australia; University of Genova, Italy

## Abstract

An international effort is underway to establish a representative system of marine protected areas (MPAs) in the Southern Ocean to help provide for the long-term conservation of marine biodiversity in the region. Important to this undertaking is knowledge of the distribution of benthic assemblages. Here, our aim is to identify the areas where benthic marine assemblages are likely to differ from each other in the Southern Ocean including near-shore Antarctica. We achieve this by using a hierarchical spatial classification of ecoregions, bathomes and environmental types. Ecoregions are defined according to available data on biogeographic patterns and environmental drivers on dispersal. Bathomes are identified according to depth strata defined by species distributions. Environmental types are uniquely classified according to the geomorphic features found within the bathomes in each ecoregion. We identified 23 ecoregions and nine bathomes. From a set of 28 types of geomorphic features of the seabed, 562 unique environmental types were classified for the Southern Ocean. We applied the environmental types as surrogates of different assemblages of biodiversity to assess the representativeness of existing MPAs. We found that 12 ecoregions are not represented in MPAs and that no ecoregion has their full range of environmental types represented in MPAs. Current MPA planning processes, if implemented, will substantially increase the representation of environmental types particularly within 8 ecoregions. To meet internationally agreed conservation goals, additional MPAs will be needed. To assist with this process, we identified 107 spatially restricted environmental types, which should be considered for inclusion in future MPAs. Detailed supplementary data including a spatial dataset are provided.

## Introduction

The high-latitude Southern Ocean, south of the Polar Front, is globally significant for conservation due to its unique species and environments. The presence of high levels of endemism shows it to be a distinct ‘realm’ within the Earth’s oceans [1]–[3]. The region is remote and has been altered less by human pressures than most other parts of the world [4]. However, exploitation has occurred across all its major marine ecosystems [5]. This has prompted the establishment of international agreements, in particular, the Convention on the Conservation of Antarctic Marine Living Resources (CCAMLR) to conserve the biodiversity while, among other roles, managing exploitation of marine living resources in the region [5]–[7].

An international effort is underway to establish a representative system of marine protected areas (MPAs) for the Southern Ocean and CCAMLR has the primary responsibility for developing this system [8], [9]. CCAMLR aims to complete this task to meet marine protection objectives set by The United Nations World Summit on Sustainable Development [8]. CCAMLR has identified the development of a broad-scale classification of the Southern Ocean and a finer-scale, classification of its biodiversity as necessary to achieve this aim [10]. Our aim is to complement this process by identifying the areas where benthic marine assemblages are likely to differ in near-shore Antarctica and the Southern Ocean. We achieve this by using a hierarchical spatial classification of the benthic environment and assessing the extent to which the existing system of MPAs encompasses the benthic diversity.

### Developing a hierarchical classification of the benthic environment in the Southern Ocean

Regional classifications are important to MPA planning and aim to identify biogeographic patterns by spatially subdividing an area, such that assemblage compositions within each subdivision are expected to be homogeneous relative to adjacent regions [2], [11]. Classifications aim to separate different assemblages and, in particular, identify areas within which species that are endemic or have restricted ranges will reside. Importantly, this process also aims to identify ecological separation (poor connectivity) for species common across assemblages. For example, some widespread species show genetic differentiation suggesting that gene flow is restricted between isolated sub-populations [12]–[15]. Therefore, at a finer-scale, higher heterogeneity is likely to be present than is often considered in the historical biogeography in the region e.g. [16]. The focus of the classification developed here is to identify possible general patterns of assemblages and of more restricted benthic species and populations at a circumpolar scale.

Physical environmental data can be used as surrogates for biodiversity where biological data are scarce [17], [18]. Ideally, the process of subdivision would be done largely, if not entirely, on the basis of biological data. However, biological data paucity is prevalent across marine ecosystems globally leading to a necessity to use surrogates [19], [20]. On a circum-Antarctic scale, biological data are limited, particularly for the Amundsen Sea, eastern Antarctica, under ice shelves and below 1500 m [21]–[23]. While this paucity of biological data introduces uncertainty into regionalisation or MPA planning processes, environmental data can be used to assess the similarity or differences between regions for the purposes of these planning processes [17], [18]. In fact, additional biological data does not always greatly increase the efficiency of protected area planning [24].

When utilizing physical environmental data in this way, a classification method should be chosen that enhances the biological relevance of the results, for example by incorporating known relationships between the physical environment and biodiversity. Recent research within the Southern Ocean has primarily focussed on using statistical clustering methods to identify areas with relatively dissimilar attributes, examples include: [11], [25], [26]. This approach has particular utility when synoptic data are available on abiotic factors that are known to be drivers or correlates of biological patterns but few data are available on actual biological patterns [27]. Where the relationships between the distribution of benthos and abiotic factors can be reasonably inferred, they can be manually incorporated to delineate regions rather than relying on an unsupervised classification e.g. clustering, [11]. For instance, the continental shelf usually supports distinct species assemblages when compared to the deeper ocean [22], [28], [29]; this has also been observed on seamounts [30]. Furthermore, the utility of biogeographical classifications to MPA planning is improved by a hierarchical (nested) classification, which shows the subdivision at increasingly finer scales [2], [20]. This approach was used by Spalding et al. [2] and Last et al. [31] to identify distinct regions based on taxonomic composition and the degree to which separation might occur as a result of patterns of dispersal, life histories of taxa, and isolation.

While a circumpolar classification of the pelagic environment has been completed and adopted by CCAMLR [25], no equivalent, accepted benthic bioregionalisation currently exists [26], although there are sufficient data to develop one. Databases of molluscs, bryozoans and zooplankton are being compiled and analysed, albeit they are limited in their geographic extent [1], [32]–[35]. The SCAR-MarBIN database is improving data availability at a circumpolar scale [36]. The relationships between depth, geomorphology and species distributions have been investigated [28], [37]–[39]. Furthermore, the connectivity between populations has been explored using genetics [14], [40], [41]. The Biogeographic Atlas of the Southern Ocean, when published, will contribute to bringing together much of this research (see www.atlas.biodiversity.aq). However at the time of writing, no synthesis of this recent research exists for the Southern Ocean.

**Figure 1:**
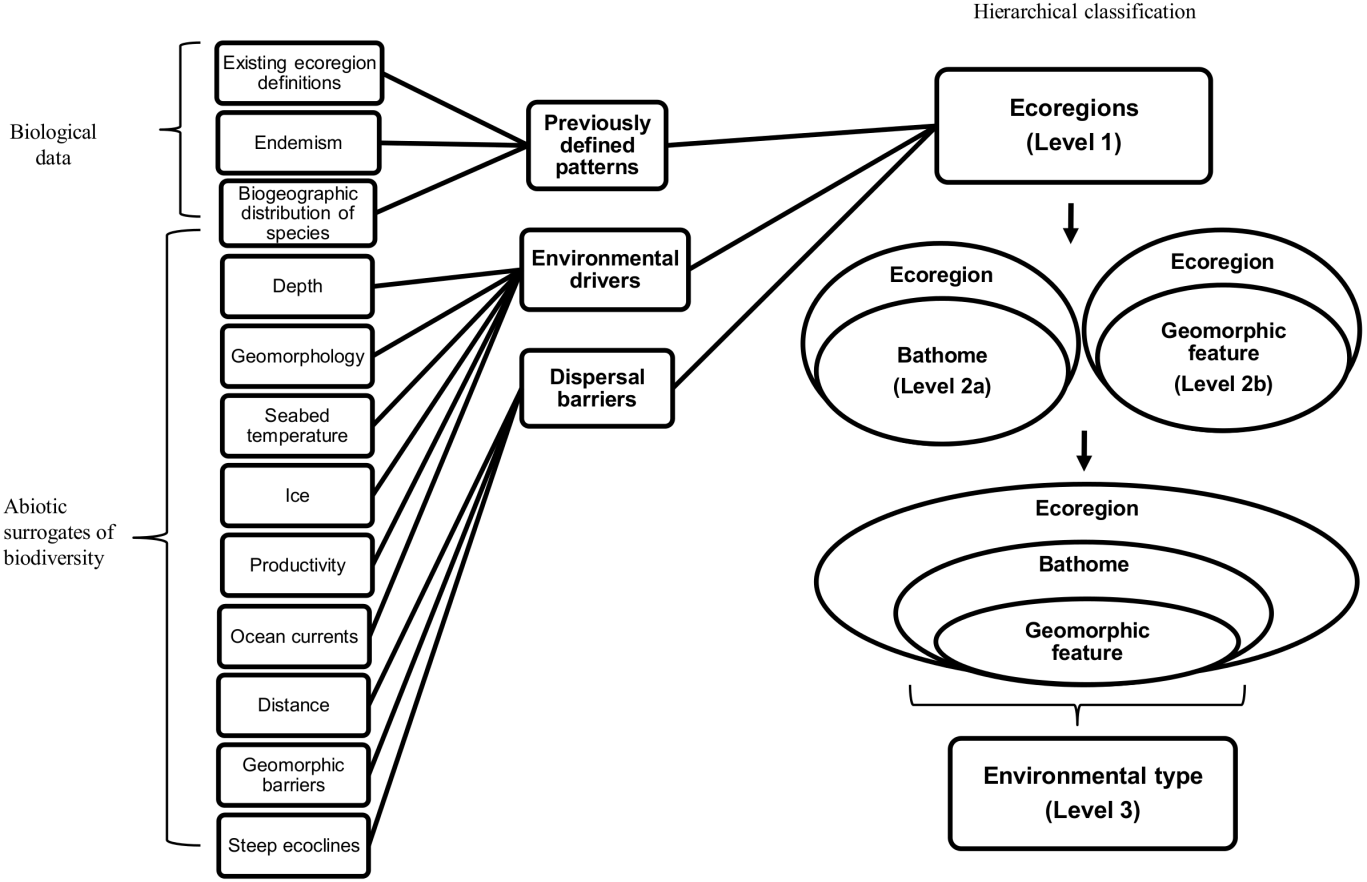
The framework used to classify ecosystems within the Southern Ocean. Ecoregions were defined on the basis of important environmental drivers and their potential to prevent dispersal. Biogeographic patterns identified in the literature were also incorporated. A hierarchical approach was then applied within each ecoregion, nesting the two main habitat types (geomorphic features within bathomes) to identify environmental types.

Here we present a circumpolar hierarchical classification of the benthic environment based on Last et al., [31] and intended for general use in spatial planning and management. The classification includes the identification of ecoregions, bathomes and environmental types, the latter of which are based on geomorphic features. Similar to the Delegations of Australia and France, [17], ecoregions were delineated by accounting for recent biogeographic research, patterns of endemism and the influence of environmental drivers as potential barriers to dispersal (Fig. 1). Environmental types represent the lowest order in the hierarchy and we used these to assess the representativeness of currently designated MPAs. We also identified environmental types which have restricted distributions and are probable locations for the focus of future MPA design.

### Environmental drivers

Environmental drivers are the physico-chemical processes and other factors that set the habitat conditions and influence the distribution and abundance of taxa, including their connectivity between similar habitats. Reduced connectivity can give rise to divergent evolution of similar taxa. Two major environmental drivers are depth and geomorphology [28], [29], [42]–[44]. Other important drivers include seabed temperature, icebergs and sea ice coverage, sea-surface productivity and ocean currents [45]–[48].

#### Depth

The Southern Ocean contains a smaller proportion of depth-restricted species than other oceans, probably due to the presence of a deeper shelf, deep-water formation (Antarctic Bottom Water), lack of thermocline and the intermittent glaciations of the shelf, creating less difference between environments on the shelf and the deep sea [49]–[51]. Wide depth ranges in Antarctic species have been typically associated with survival in deep-sea refuges e.g. [49]. That is, sub-populations of eurybathic shelf species may have endured past glaciations on the slope or deeper, which could have then served as a potential source for shelf (re-) colonization [28], [49], [52]. Thatje et al. [50] suggested, that life on the Antarctic continental slope during the last glacial maximum may have been extremely difficult due to turbidity currents cascading down the slope. Sedimentation processes would not have occurred everywhere though, and were probably more pronounced along canyons compared to adjacent flanks and plateaus leaving some scope for slope fauna to survive [28]. Limited depth distributions of shelf species, on the other hand, could serve as evidence for survival through glaciations in ice-free refuges on the continental shelf [53].

Many benthic species do have restricted depth ranges and depth-related factors can be strong barriers to dispersal of benthic species [37,38,54,55,56 and see ‘Barriers to dispersal’ section]. For instance, the octopus genus *Paraledone,* the echinoid genus *Sterechinus*, the ostracod genus *Macroscapha* and the amphipod genus *Eurythenes* show niche separation by depth [40], [57]–[59]. This depth partitioning gives rise to different assemblage structures in different depths [60], the ranges of which can be characterized as bathomes [31]. Furthermore, molecular studies show widely distributed species often represent species complexes, with each individual species having a much more restricted range size. For example, isopods: [56], [61], [62]; crinoids: [13]; and ophiuroids: [55].

#### Geomorphic features

Geomorphic features are a classification of the seabed based on its surface morphology, and have been shown to be significant for delineating benthic habitats [63]. The features used here are meso-scale habitat features related to the sedimentary habitat along with the near-bottom conditions of the ocean environment [31]. Major habitat characteristics include the availability of hard-rock surfaces and the erosion or deposition of sediment along with their physical attributes [42], [64]. For example, Antarctic shelf depressions eroded during glacial maxima now have low currents and fine sediments, providing appropriate habitats for mobile deposit feeder and infaunal communities [46], [64], [65]. The species rich coral-sponge communities found within some shelf-cutting canyons are postulated to be due to an abundance of food contained in channelled water sourced from productive shelf regions [43], [66]. Elsewhere, canyons are shown to alter current flow causing upwelling and mixing which are attributed to enhanced local biological systems [67]. Seamounts have also been shown to be important for biodiversity and can have high endemism levels [30], [68]–[70]. Hydrothermal vents are shown to contain distinct species and unique community structures [71]. Research in the Weddell Sea shows a clear distinction in faunas of the shelf, slope and abyss [28], [53]. However, this may not be the case in all regions and for all species. For example, such patterns have not been found to exist within the Scotia Sea [28].

#### Seabed temperature

Seabed temperature has a major influence on the ecology of Antarctic benthic fauna; its spatial and temporal variability contributes to determining the biogeography of those fauna; and the composition of assemblages [27], [45]. Seabed temperature is suspected to constrain the migration of benthic fauna and may lead to genetic variation and eventual speciation [27]. This hypothesis is supported by recent research on the Antarctic sea spider, Nymphon australe and the octopus genus, Pareledone [12], [72]. Temperature can be biologically meaningful even where the spatial and temporal variation is narrow [73].

#### Ice

Ice regimes are a key structuring element in the ecology of the Antarctic benthos [48], [74], [75]. Icebergs scour the shelf seabed, impacting the distribution of benthic habitats and the diversity of assemblages [39], [42], [76]. Areas under glacier tongues and fast ice are deprived of inorganic sedimentation leading to dominance of sessile suspension feeders [76]. Benthic habitat conditions can also be affected by the influence of ice on light availability, water currents, temperature, biogeochemistry, algal growth and productivity [42], [48], [77]–[80]. These conditions may be modified depending on the concentration and duration of the sea ice [78], [81].

#### Productivity

Sea surface productivity provides a vital food source to the benthos [82] and the inter-annual reliability and magnitude of productivity blooms are likely drivers of total productivity in the benthic system [75]. Phytoplankton blooms are highest (i) where frontal activity has created an upwelling of nutrient-rich water, (ii) down-stream from iron-rich landforms and (iii) within the ice-melt zones and polynyas [83]–[87]. Polynyas are regions of reduced ice concentration surrounded by heavy pack ice. Feeding especially within the near shore benthos can be seasonal [88]–[90]. Many benthic ecological processes though, appear not to be coupled with the summer sea surface productivity bloom due to the presence of persistent sediment food banks and the influence of bottom circulation on sediment distribution [46], [75]. However, interaction between the sea surface and benthos can occur through sedimentation of organic matter from the surface [75], and through trophic transfer from herbivores at the surface to delivery to the benthos [91]. For example, in the latter case, salps feed on phytoplankton then vertically migrate to the demersal zone where they form the near-exclusive prey species of benthic octocorallian polyps [47]. In the former case, macrobenthic biomass in muddy sediments of the deep Antarctic shelf is correlated with regional primary production and sea-ice duration [75]. Similarly, organic matter is transferred within sinking water generated from rejected brine during ice formation and is suspected to act as an important source of food for benthic species creating locations of high species richness in deep-sea areas traversed by this organically laden bottom water [22], [51].

### Barriers to dispersal

The geographic distribution of a species and genetic connectivity between subpopulations is linked to the ability of individuals to disperse away from their parents. This ability will be constrained by dispersal barriers including the distance between suitable habitats, geomorphic barriers and steep environmental clines. Long-distance dispersal in benthic organisms is confined to passive oceanic transport of adults or via turbidites (i.e. rafting), and transport during pelagic larval stages [92].

Habitats may support different species assemblages where the distance between suitable habitats is greater than the distance over which a species can disperse. While the dispersal distances for most species in the Southern Ocean are unknown [93], it is likely that the dispersal range for many brooding species will be restricted; brooding species protect and feed their offspring without a pelagic larval stage [50]. Within the Southern Ocean, benthic communities have more species with either lecithotrophic (non-feeding) larval development or brooding than species with planktotrophic (feeding) larvae [50]. While the strong, circumpolar currents of the Southern Ocean may assist the dispersal of some species with pelagic larval stages [94], [95], predation, parasitism and starvation will constrain the probability of moving great distances, especially for lecithotrophic larvae which have a limited food source [94], [96]. Furthermore, strong genetic variation has been shown in some widely dispersive species suggesting higher heterogeneity at a finer-scale [12]–[14].

Geomorphic barriers, steep ecoclines and ocean currents can also cause ecological and genetic differentiation. For example, differences in seabed temperature across relatively short distances can cause genetic differentiation within a species [12]. Similarly, genetic variation can occur through the inhibition of adult migration by geomorphic features [97]. Ocean current and frontal systems, depth-related factors as well as habitat fragmentation can also be strong physical barriers to gene flow [14], [81], [94], [95], [98], indicating that the ecological dynamics of populations can be spatially restricted. Examples include studies comparing shelf dwelling benthic diversity between the Antarctic Peninsula and Scotia Arc [14], [55]. Changes in oceanographic conditions are also important barriers to dispersal in the open ocean [14], [29], [94].

### MPA Assessment

A gap analysis can identify where additional MPAs are required to ensure that all ecosystems and biodiversity are represented within a system of MPAs [99]. There are very few MPAs within the Southern Ocean. The largest is south of the South Orkney Islands [100], [101]. Other MPAs include those within the Australian, French, Norwegian and South African territories and some small areas protected under the Environmental Protocol to the Antarctic Treaty (Madrid Protocol).

We use the fine-scale (<100’s of km’s) environmental types as surrogates for different assemblages of biodiversity to assess the representativeness of existing MPAs. A system of MPAs that captures each environmental type is assumed to also capture the range of biodiversity these habitats represent. We also identify those environmental types that have distributions restricted by geographic area and location. The environmental types within these restricted locations are not necessarily more important than others ecologically. However, they tend to be the focus of MPA selection since there are limited spatial options for protecting the biodiversity for which they are a surrogate.

## Materials and Methods

The region that is managed by CCAMLR defined the study area. The northern boundary of this region is a line approximating the location of the Polar Front [25]. The southern boundary was defined as the northern edge of the permanent ice shelf of the Antarctic continent.

### Data

Table 1 provides an overview of the circumpolar datasets used in this study, their spatial and temporal resolutions along with their data sources. Additional regional information on seabed currents was also geo-referenced including: [102]–[105]. The spatial resolutions of the environmental data sets varied but were generally around 10 km with the exception of the seafloor temperature. However, only the depth and geomorphic data were directly utilized to define the environmental types, with the remaining data used to elucidate general environmental patterns. Thus, the effects of these scale mismatches were minimal.

**Table 1:** Circumpolar datasets used within the classification.

Data	Spatial resolution	Temporal resolution	Source
Depth	1minute	Not applicable	Smith and Sandwell, [118]
Geomorphology	1–12 km	Not applicable	O’Brien *et al*. [64]
Seafloor temperature	1 degree	Annual mean over allyears available in the WorldOcean Atlas 2005 [127]	Clarke et al. [27]
Sea surface chlorophyll-a	9 km	Mean values for each australsummer season (20^th^ Dec to 20^th^ March)for years 1998–2010	Feldman and McClain, [128]
Sea ice concentration	6.25 km	The proportion of the year where sea ice concentration was atleast 85% derived from daily estimates during the1^st^ January 2003 to 31^st^ December 2009	Spreen et al. [129]
Frontal systems	20 km	Annual mean calculated across 1992–2007	Sokolov and Rintoul, [130]

The datasets were not modified except for sea-surface chlorophyll *a*. For this dataset we used a time series to identify areas of consistently high and low productivity. For each available season 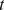, the mean summer concentration in each grid cell 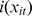 was transformed to an anomaly 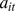 by subtracting the seasonal mean 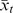 for the entire study area (using log-transformed values):
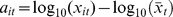
To distinguish areas of intermittent high productivity from those of consistent but moderate to high productivity, these anomalies were further transformed giving an index 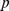 of the consistency of productivity from season to season [17]:
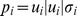
Where 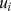 and 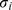 are the mean and standard deviation of 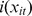 calculated over all available seasons 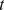.

We also developed two datasets of proposed and existing MPAs. The MPAs proposed from MPA planning analyses current at September 2013 were; 1) The seven MPAs proposed from a study of Eastern Antarctica south of 60°S and between 30°E and 150°E [17] and; 2) The MPAs proposed from a study of the Ross Sea region south of 60^o^S and between 150^o^E and 150^o^W [106]. Existing MPAs that have been designated or acknowledged by CCAMLR were included. These MPAs included; 1) The South Orkney Islands Southern Shelf MPA within the high seas [100]; 2) The Kerguelen and Crozet Islands within the French EEZ [107]; 3) The Prince Edward and Marion Islands within the South African EEZ [108]; 4) The Heard and MacDonald Islands within the Australian EEZ; 5) A twelve nautical mile buffer around Bouvet Island for the Bouvetøya Nature Reserve [109]; 6) One nautical mile exclusion zones around Vulnerable Marine Ecosystems notified in accordance with CCAMLR conservation measures that regulate bottom fishing (specifically measures 22-06 and 22-07) [110]–[112] and; 7) Marine areas listed as designated under the Environmental Protocol to the Antarctic Treaty and which have management plans (available at www.ats.aq) approved by CCAMLR in accordance with Antarctic Treaty Consultative Meeting decision 9 in 2005 [113], [114].

### Analysis

Broad spatial boundaries, termed benthic ecoregions, were identified according to the primary environmental drivers and dispersal barriers that drive the distribution of Southern Ocean benthic biodiversity. Within these ecoregions, biodiversity patterns are further driven by finer-scale spatial processes. Therefore, we applied a hierarchical framework based on Last et al. [31] to identify each environmental type. Bathomes (broad-scale depth classes) were nested within ecoregions, and geomorphic features were nested within bathomes. Each environmental type is therefore a unique combination of geomorphic feature, bathome and ecoregion. The nesting of geomorphic features within bathomes avoids the potential for false within-class homogeneity that can arise if such features are not modified by depth [115]. The analysis was performed using both manual and automated processing within a geographic information system and is further described in the following six sections. Further detail of the analysis is provided as supplementary information including ecoregion boundary descriptions (Text S1), the spatial coverage of each level of the classification (Table S1) and a description of the methods used to generate geomorphic features (Text S2).

#### Benthic ecoregions

The primary environmental drivers used to identify benthic ecoregions were depth, geomorphology, seabed temperature, sea ice concentration and chlorophyll-a as a proxy for productivity. The three major types of barriers to dispersal we used were; 1) the distance between potential habitat types (i.e. bathomes and geomorphic features); 2) large geomorphic barriers and; 3) presence of steep ecoclines in temperature, ice or productivity. The influence of both frontal and seabed currents were considered as either dispersal barriers or assisting connectivity between habitats (as further described in Text S1).

We initially delineated the distinct environments of the continental shelf and slope from the deeper ocean and then applied bathomes based on depth-species relationship studies to further account for the depth structuring of benthic assemblages. The 4000 m bathymetric contour was the most appropriate to generally separate the geomorphic features associated with the shelf and slope of the Antarctic continent from the geomorphic features associated with the abyssal ocean. Exceptions to this contour included parts of the East Indian Abyssal, Pacific-Antarctic Ridge, Kerguelen and Pacific Basin ecoregions which were adjusted to better capture geomorphology. Where the structural slope of the Oates ecoregion extends past the 4000 m contour in the Indian Ocean sector, the boundary of the structural slope was used in preference to the 4000 m contour. The boundary between the Kerguelen ecoregion and the Antarctic continent was defined by the southern limit of the contourite drift (i.e. a geomorphic feature), the slope of the broader Kerguelen Plateau (i.e. including Banzare and Elan Banks) and the canyon at 77.5^o^E, 61.5^o^S. Similar to Spalding et al [2] we included the seamounts and the seamount chain near to 120^o^W within the Amundsen ecoregion.

Where possible, previously defined ecoregions were reviewed and incorporated especially: [1], [2], [17], [25], [32], [33]. These studies have focussed primarily on the Antarctic continental shelf and slope where more data are available. The previously defined boundaries were upheld if the ecoregion contained levels of endemism greater than 10% as reported by Linse et al. [32] or Griffiths et al. [1]. In each case, these boundaries were adjusted to better reflect the distribution of bathomes and geomorphic features. For instance, geomorphic features can act as barriers to dispersal for some benthic species leading to genetic distinctions [97]. Therefore, if deemed more appropriate, features such as canyons were used to replace the previously defined boundaries. Also, where a previously defined boundary divided a feature such as a canyon, it was redrawn to capture the entire feature. Within the deeper ocean, ecoregion boundaries were defined by considering the effect of structuring agents on patterns of biodiversity.

For areas without previously defined ecoregions, we initially identified where areas of similar habitat types (predominantly bathomes and geomorphic features) were separated by more than 200 km. This distance is based on the general global maximum of 200 km for dispersal distance across multiple marine species even where uncertainly is accounted for [116], [117]. Spatial separation is assumed to increase the likelihood of areas of the same habitat type supporting genetically dissimilar species due to isolation and therefore contributes to the separation of ecoregions. All data layers were then used to corroborate or refute the validity of these regions defined by the separation of similar types of areas by the minimum distance. For instance, where large geomorphic barriers, including ridges, could contribute to separating areas of deeper habitats this would be seen as an ecoregion boundary between these two areas of habitat. Similarly, rapid changes in seabed temperature, sea ice, persistent productivity and ocean currents were also considered as signifying potential barriers to dispersal or where an ecoregion boundary could be most meaningful ecologically.

#### Bathomes

Bathomes are broad-scale depth classes. They were derived by dividing the region into different depth divisions based on bathymetry data which are frequently updated from satellite altimetry and ship depth soundings [17], [118], [119]. The boundaries were based on the depths at which there are expected to be rapid transitions in assemblage composition. We identified those depths based on available depth-species relationship studies within the Southern Ocean. Depth-species relationship studies are summarised in Table 2.

**Table 2:** Benthic bathomes and the ecological and biological events used to define them.

Bathome (m) Depth Range	Ecological and Biological Events. Unless otherwise stated, the Bivalvia, Gastropoda, Isopoda and Polychaeta taxa discussed only include the species studied in Brandt et al. [37].
0–100	Seaweed availability limits depth of herbivores [131].
	Polychaete species richness is highest.
	12 paramunnid species and 8 genera of Isopod family Paramunnidae are restricted to the top 100 m (S. Kaiser, unpublished data).
	4 pycnogonid species have been recorded exclusively from the top 100 m [132]
100–200	200 m is likely to be the maximum extent of the influence of wave action and sunlight penetration [64].
	Polychaete species richness begins to rapidly decrease.
	Chlorophyll concentration is generally negligible below 200 m [83].
200–500	High gastropod species richness at approximately 200 m–300 m.
	The end of the depth range of *Channichthys rhinoceratus* which is located in depths less than 200 m [38].
	200–500 m is the depth range of *Champsocephalus gunnari* [38], [133].
	*Zanclorhynchus spinifer* depth range begins [38].
	500 m is the approximate maximum depth of scouring by contemporary ice bergs [42], [43], [134].
	*Lepidonotothen squamifrons* exhibits some areas of high density close to the 500 m isobath [38].
	Species of the isopod family, Santiidae are only found in the top 500 m.
	Most hydroid species only occur above 500 m [135]
500–1000	Upper slope mollusc assemblage is present between 400 and 800 m [136].
	Many echinoid species can only be found in the top 1000 m see suppl. Material of [28] and [137].
	Many bryozoan species restricted to the top 1000 m [53].
	Seven fold less gastropod species than at 200–300 m.
	The bivalve families Arcidae and Vesicomyidae can now be found. Condylocardiidae, Nuculidae, Hiatellidae and Erycinidae are no longer found.
	The Polychaeta families; Dorvilleidae, Chaetopteridae, Lacydoniidae, Pectinariidae and Spintheridae are no longer found.
	Much lower densities of fish than depths <500 m [133]. Four fish-depth ranges begin (*Bathyraja eatonii, Bathyraja irrasa*, *Alepocephalus* cf. *Antipodianus* and *Etmopterus* cf. *granulosus*) [38]. *Z. spinifer* depth range ends [38]. *Sterechinus neuymeyeri* depth range is between 0–810 m [40].
	Most pycnogonid species (63.8%) occur above 1000 m [132].
1000–1500	Bivalves, gastropods and polychaete diversity decreases from the shelf to the slope then stabilises at low numbers. There is no observed replacement of diminished shelf polychaete species from the slope and rise community (A. Brandt, unpublished data). The bivalve famillies Arcidae (*Bathyarca sinuata*) and Vesicomyidae (*Vesicomya sirenkoi*) can now be found regularly down to the abyss.
	The number of isopod species increases. Isopod families, Macrostylidae, Ischnomesidae and Haploniscidae are mostly found below 1000 m depth (with the exception of a few species found at shallower depths).
	Chaetognaths become much less abundant [138].
1500–2000	The gastropod families; Acetonidae, Cancellariidae and Cerithiidae are no longer found.
	The bivalve families; Pectinidae, Lyonsiidae and Astartidae are mostly only found down to 2000 m.
	Sequenziidae (gastropod) and Cyamiidae (bivalve) are located in depths shallower than 1500 m and deeper than 2000 m.
	ANDEEP samples showed isopod species typical of the shelf to penetrate to a depth of 1500–2000 m [139].
	Presence of a lower slope mollusc assemblage between 800 to 2000 m [136].
2000–3000	Depth band in which the gastropod family, Marginellidae is located (also located at depths <800 m). Also, the family Propilidiidae can now be found.
	The shelf inhabiting bivalve family Philobryidae is generally found in shallow water (<2000 m). However, the genus *Adarcnarca* can be found deeper than 2000 m.
	Isopod species richness continues to increase. The Isopod family, Austrarcturellidae is no longer found and Gnathiidae is found above and below this range.
	The main depth range of the polychaete family Pectinariidae (beside <500 m). Also, the Sabellariidae begin to be located (beside <200 m) and Eunicidae are no longer found.
3000–4500	Isopod species richness is highest within this depth range.
	Number of gastropod and polychaete species per depth begins to decrease.
	Echinoid species *Echinosigra amphora* and *Pourtalesia debilis* only occur between 3000 m and 4000 m [137].
	Gastropod species richness becomes extremely low and the gastropod families, Volutomitridae, Trichotropidae, Pleurobranchiidae, Fissurellidae are no longer found.
	The Isopoda families, Bopyridae and Stenetriidae are no longer found.
	The polychaete families, Pectinariidae are no longer found and Apistobranchidae end close to 3000 m.
	The bivalve families, Malletiidae and a species of Kelliidae are found deeper than around 3000 m.
	Only six bryozoan species occur below 3000 m [53].
	Polychaetes are now a deep-sea assemblage composed of genera considered typical within the deep sea worldwide. This shift begins at approximately 2500–3000 m and extends onto the abyssal plain [22]
4500+	Isopod and polychaete species richness drops rapidly. However this could also be due to sampling bias, as there are very few samples deeper than 5000 m. Isopoda families: Cirolanidae, Dendrotionidae, Gnathiidae, Paramunnidae and *Xostylus* incertae sedis are no longer found within samples.

#### Geomorphic features

We identified geomorphic features based on O’Brien et al. [64] (see Table 1 and Table 3). This dataset, however, did not cover all areas within the study region due to the absence of data in areas of persistent ice cover. All areas not covered were located on the Antarctic continental shelf and we classified them as ‘Shelf’. We also modified some features. ‘Fracture zone cliffs’ and ‘Canyon axes’ had been mapped as lines by O’Brien et al [64]. We converted ‘Fracture zone cliffs’ to areas through interpretation of the bathymetry contours with contours located close together considered part of the cliff. ‘Canyon axes’ were converted to areas by applying a 5 km buffer around the axes to capture both the canyon and its surrounding zone of influence. We deemed a 5 km buffer to be the most appropriate distance to ensure connectivity within dendritic canyon systems and discontinuity between single channelled canyon systems. The geomorphic features classified by O’Brien et al. [64] as ‘Wave affected banks’ are identical to their ‘Shelf banks’, but at depths of less than 200 m. This depth modification of geomorphology is captured by our nested hierarchical analysis and therefore these features were combined and renamed ‘Banks’. The ‘Coastal terrane’ of South Georgia Island was reclassed as ‘Island coastal terrane’ to be consistent with other island areas. The Antarctic ice shelf including areas under floating ice tongues termed ‘ice shelf cavities’ were excluded.

**Table 3:** The geomorphic features of the Southern Ocean classified according to the attributes of the seabed surface substratum.

Geomorphic class	Name (map code)	Definition adapted from O’Brien et al. [64]
Continental Shelf and related features	Bank (2)	Broad shallow regions typically at depths of 100–200 m. The boundary between the shelf bank and shelf depressions is set at a depth of around 500. Banks within both the South Georgia and Kerguelen Plateau ecoregions were classed as Oceanic Shallow features.
	Coastal (Rugged)Terrane (5)	Region of varying seafloor type and depth ranges along rugged coastlines
	Cross Shelf Valley (7)	Shelf depressions that are connected to the shelf edge via valleys.
	Shelf (22)	Unclassified regions within the continental shelf region.
	ShelfDeeps-Depressions (23)	Shelf region generally deeper than 550 m with closed contours.
	Volcano (28)	Distinguished from other islands and seamounts, where volcanic processes directly impact the marine environment. Mapped volcanoes within the Southern Ocean all occur on the shelf.
Oceanic Shallow features	Island Arc (9)	Islands formed from bow-shaped volcanic ridges adjacent to subduction zones.
	Island Coastal Terrane (10)	Similar to coastal (rugged) terrane representing a zone of high variability around islands.
	Margin Ridge (13)	Ridges formed from igneous or basement intrusions along the continental margin and protruding hundreds of meters above the (abyssal) sediment plain.
	Marginal Plateau (14)	Areas of relatively level sea floor at mid depth extending from continental margins and separated from the shelf by a saddle.
	Plateau (16)	Relatively flat regions elevated above the surrounding sea floor by more than a few hundred meters.
	Ridge (18)	Elongate ridges that may extend from a plateau or other feature.
	Seamount Ridges (20)	Elongate ridges that protrude hundreds to thousands of meters above the surrounding sea floor. Their shape has the potential to influence deep current activity.
	Seamounts (21)	Roughly circular areas which rise above the surrounding sea floor by at least 1000 m.
Slope and related features	Canyon (3)	A relatively narrow, deep gully with steep sides. Axes were traced along landward contour inflection points, particularly in the shelf edge region.
	Lower Slope (11)	Region on the continental slope of reduced gradient with a lower limit where slope canyons are no longer obvious (around 2500–3500 m below sea level).
	Plateau Slope (17)	Broad regions sloping from the margins of large plateaus to the surrounding deep ocean floor.
	Structural SlopeRegion (24)	Low relief topographic features formed from underlying structures, such as basement protrusions, that extend beyond the lower slope.
	Trough Mouth Fans (26)	Broad aprons of smooth to slightly gullied sediment on the Upper Slope extending from the shelf break to 2500–3000 m water depth.
	Upper Slope (27)	Seaward dipping slope extending from the continental shelf break which is defined as the position at which the rate of change in slope gradient is at a maximum.
Abyss and related features	Abyssal Plain (1)	Extensive, flat, gently sloping or nearly level region of sediment covered seafloor at abyssal depths.
	Cliff (4)	Very steep or near vertical features normally occurring at major crustal fractures or on the sides of glacial valleys on the shelf and are likely to expose hard substrates.
	Contourite Drift (6)	Sediment mounds constructed by strong bottom currents that rise gently above the surrounding sea floor.
	Fracture Zone (8)	Major oceanic crustal fracture zones.
	Mid-Ocean Ridge Rift Valley (12)	Elongate troughs created by seafloor spreading, extending several hundred meters below the rift shoulders and containing hydrothermal vents.
	Ocean Trough (15)	Closed elongate depressions (in the ocean floor) more than 4500 m deep and hundreds of kilometres long, generally associated with fracture zones.
	Rugose Ocean Floor (19)	Relatively young oceanic crust with rugged features protruding through the sediment.
	Trench (25)	Arcuate depressions, typically at depths of more than 5000 m and reaching 6000 m in places, formed by subduction of oceanic crust.

#### Nesting data layers

Bathomes were nested within ecoregions, and geomorphic features were nested within bathomes to identify environmental types except for the following seven geomorphic features. Four geomorphic features ‘Shelf’, ‘Cliff’, ‘Coastal terrane’ and ‘Island coastal terrane’ were not further modified by depth. Seamounts and Seamount ridges were classed by only their shallowest bathome. Canyons were classed by whether they commenced on the shelf or slope. The environmental types were reviewed to minimise the potential for false heterogeneity. Environmental types with a very small area or restricted distribution were checked and dissolved into the surrounding environmental type if deemed invalid. For example, small patches of shallow rugose ocean floor within the South Atlantic ecoregion initially appeared as a restricted or rare environmental type. Upon review, this environmental type was found to be a processing artefact caused by the presence of adjacent seamounts. It was therefore eliminated by reclassing it as the most appropriate surrounding environmental type.

#### Representation of MPAs

We condensed the geomorphic features into four broad classes (Table 3). The distributions of existing MPAs and environmental types were overlaid. For each geomorphic class within each ecoregion that contains an existing MPA, we calculated; 1) the proportion of area within an MPA and, 2) the proportion of the number of environmental types with at least part of their area within an MPA. The total number of environmental types and seafloor area of each geomorphic class was also calculated.

#### Restricted environmental types

We specifically identified those environmental types which had a restricted spatial distribution. We assumed environmental types to have restricted distribution if they were either small in number (3 or less) or are predominantly limited to a particular discrete location without replication within an ecoregion. Since our focus was identifying areas where additional MPAs are required to fill large gaps in representation, restricted environments were not identified within regions where proposed MPAs have been identified through fine-scale MPA planning processes. Therefore, the region east of 30^o^E to 150^o^W and south of 60^o^S was excluded from the restricted environments analysis. Instead, the proposed MPAs within these regions were compiled and incorporated [17], [106].

## Results and Discussion

### Classification

**Figure 2:**
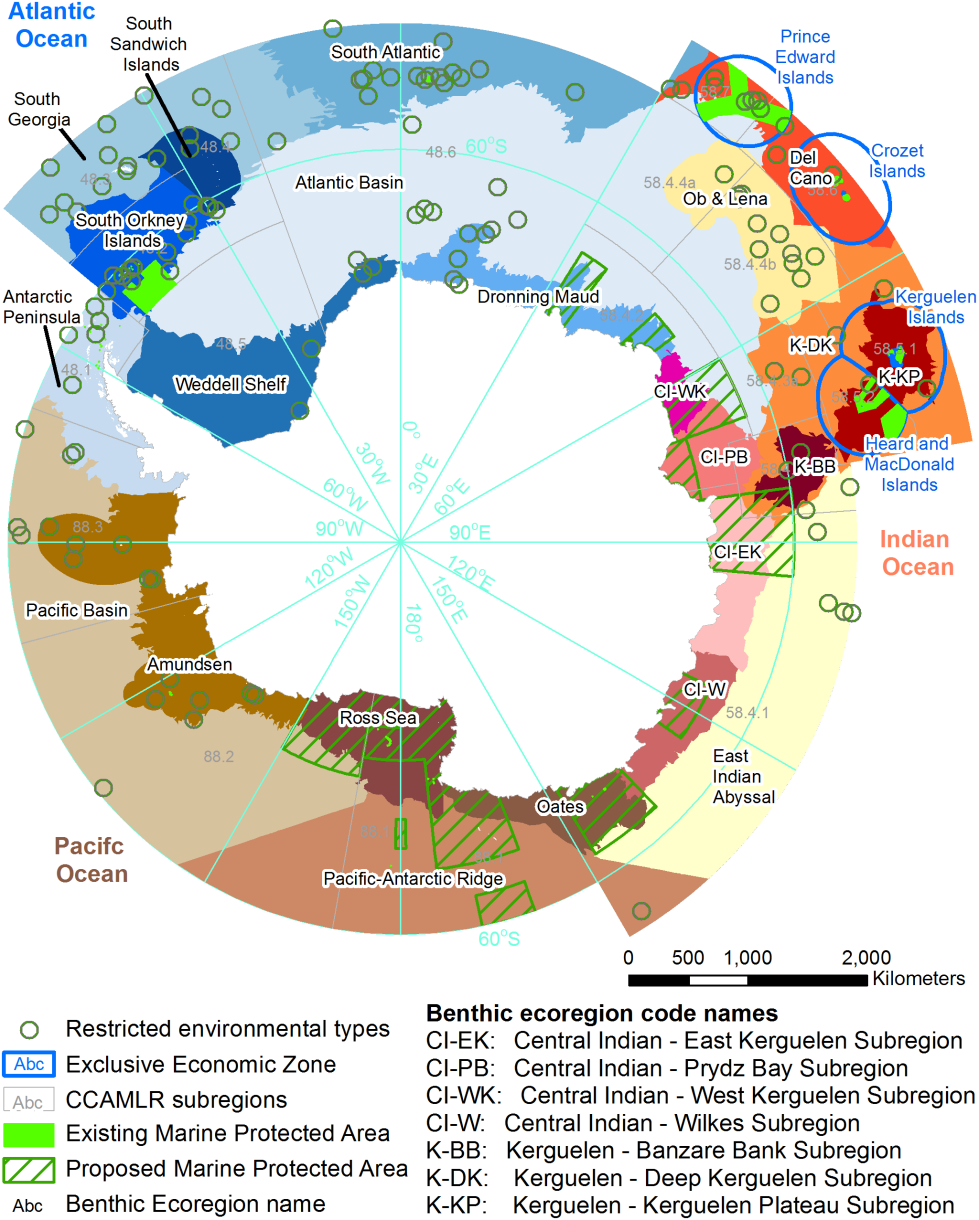
The benthic ecoregions, restricted environments and marine protected areas identified within the Southern Ocean. An environmental type is a unique combination of an ecoregion, bathome and geomorphic feature. Existing marine protected areas and regions where planning processes are underway to propose future representation, were identified. Where large gaps in existing and proposed representation were found, the locations of geographically restricted environmental types were identified. These restricted environments indicate areas of potential future marine protected area selection since there are limited spatial options for protecting the biodiversity for which these environments are a surrogate.

**Figure 3:**
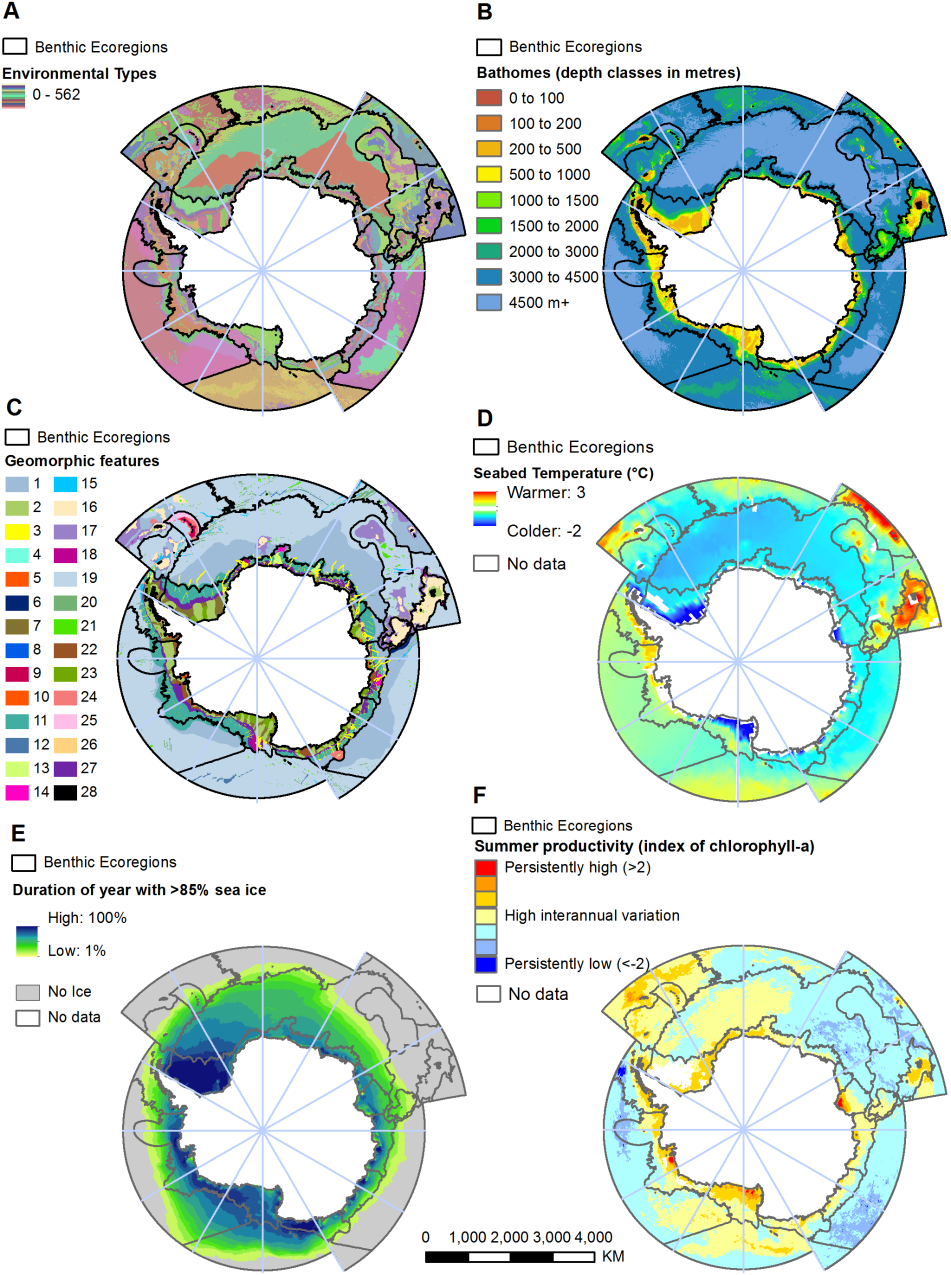
Environmental types and the bio-physical data used to drive the classification. **A)** The 562 environmental types (in colour) and ecoregion outlines (refer to figure 2 for names) broadly reflect the underlying data used within the classification **B)** Bathomes derived from bathymetry and species-depth relationships **C)** Geomorphic features; Abyssal Plain (1), Bank (2), Canyon (3), Cliff (4), Coastal (rugged) Terrane (5), Contourite Drift (6), Cross Shelf Valley (7), Fracture Zone (8), Island Arc (9), Island Coastal Terrane (10), Lower Slope (11), Marginal Ridge (12), Marginal Plateau (13), Mid-Ocean Ridge Rift Valley (14), Ocean Trough (15), Plateau (16), Plateau Slope (17), Ridge (18), Rugose Ocean Floor (19), Seamount Ridges (20), Seamount (21), Shelf (22), Shelf Deep (Depressions) (23), Structural Slope Region (24), Trench (25), Trough Mouth Fans (26), Upper (Continental) Slope (27), Volcano (28) **D)** Seabed temperature **E)** Duration of the year where more than 85% of the region is covered by sea ice **F)** High positive and negative values indicate areas of consistently high and low summer productivity respectively. Values approaching zero indicate areas that vary greatly between years.

We identified 23 benthic ecoregions and 9 bathomes (Fig. 2, Table 2). A general description of the ecoregions is provided (Table 4). Existing geomorphology mapping has identified 28 geomorphic feature types (Table 3, [64]). When these three datasets were combined within the hierarchical framework, 562 environmental types were identified (Fig. 3 and Table S1). The spatial dataset is available online (see http://dx.doi.org/10.4225/15/53A3760D4AFAA).

**Table 4:** Benthic ecoregions and their features (see Fig.2 for the location of each ecoregion).

Benthic ecoregion	General description
Amundsen	The productive shelf and polynyas of the Amundsen and Bellingshausen seas. The oceanic shallow environments of Peter I Island, De Gerlache Seamounts and the Marie Byrd Seamount group.
Antarctic Peninsula	The shallow, productive shelf of the west Antarctic Peninsula with a low duration of sea ice cover and warm seabeds relative to other Antarctic shelf areas. The island ecosystems of the South Shetland Islands. 13 endemic molluscs [11]. Greater than 10% of gastropods endemic [12].
Atlantic Basin	The very deep and very cold rugose ocean floor and abyssal plain of the South Atlantic Ocean Basin and Weddell Sea.
Central Indian-East Kerguelen Subregion	Central Indian region of East Antarctica that is influenced by the Kerguelen Plateau including downstream productivity from frontal activity across the Kerguelen Plateau [17].
Central Indian-Prydz Bay Subregion	Central Indian region of East Antarctica that contains the cold, productive waters of Prydz Bay and the Prydz Gyre which oceanographically separates the east Kerguelen and west Kerguelen Central Indian subregions [17].
Central Indian-West Kerguelen Subregion	Central Indian region of East Antarctica that is not influenced by the Kerguelen Plateau nor the Weddell Gyre [17].
Central Indian -Wilkes Subregion	Central Indian region of East Antarctica that is oceanographically separated from the Central Indian-East Kerguelen subregion [17], [140].
Del Cano	The shallow, warm seabeds in the Subantarctic Frontal Zone including the South West Indian Ridge seamounts, the Del Cano Rise and the Crozet and Prince Edward Islands.
Dronning Maud	Maud Rise and associated open ocean polynya, Astrid Ridge, Gunnerus Ridge and the canyons offshore Dronning Maud Land. Easternmost extent of the Weddell Gyre. 20 endemic molluscs (19% of documented species) [11]. 21% of documented gastropods are endemic [12].
East Indian Abyssal	The very deep and cold seabeds of the rugose ocean floor and abyssal plains of the South Indian Ocean Basin.
Kerguelen-Banzare Bank Subregion	Shallower (mostly depths between 1000 to 3000 m), warmer seabeds of the Banzare Bank, south of the frontal activity of the Fawn Trough.
Kerguelen-Deep Kerguelen Subregion	Deep (mostly depths greater than 3000 m) ocean surrounding the Kerguelen Plateau and Banzare Bank.
Kerguelen-Kerguelen Plateau Subregion	Shallower (mostly depths between 200 m to 3000 m), warmer seabeds of the Kerguelen Plateau, north of the frontal activity of the Fawn Trough.
Oates	Oceanographically separated from the Central Indian-Wilkes subregion with wind and sea ice vectors diverging at its western border [17]. The eastern border is adjacent to the Ross Sea region [2], [33], [125].
Ob & Lena	Shallow, warm seabeds in the Polar Frontal Zone, including the Ob and Lena banks and the seamounts to their east.
Pacific Basin	The very deep rugose ocean floor and abyssal plains of the South Pacific Ocean Basin which is warmer than other deep ocean basin regions of the Southern Ocean.
Pacific-Antarctic Ridge	The Pacific-Antarctic Ridge region with large extents of shallower environments of depths less than 2000 m.
Ross Sea	Very cold seabed and high sea ice duration of the productive Ross Sea. 22 endemic molluscs (11.5% of documented species) [11]. 16% of documented gastropods endemic [12].
South Atlantic	Shallower environments of the Mid Atlantic Ridge and associated seamounts.
South Georgia	Productive, shallow environments in the Polar Frontal Zone including the island ecosystems of South Georgia Island and the seamounts of the North Scotia Ridge. 65 endemic molluscs (32.7% of documented species) [11]. 15% of documented Cheilostomata endemic [12]. 13% of documented bivalves endemic [12]. 36% of documented gastropods endemic [12].
South Orkney Islands	The island ecosystems of the South Orkney Islands and the seamounts and plateaus of the South Scotia Arc, many of which underlie the Southern Antarctic Circumpolar Current Frontal Zone. 22 endemic molluscs (19.6% of documented species) [11]. 25% of documented gastropods endemic [12]
South Sandwich Islands	Highly productive island ecosystems of the South Sandwich Islands and the deeper waters of the South Sandwich Trench.
Weddell Shelf	Very cold seabed and high sea ice duration of the productive Weddell shelf, usually rather deep, ∼500 m, at places even down to 1000 m. 55 endemic molluscs (19.7% of documented species) [11]. 26% of documented gastropods endemic [12]

The ecoregion boundaries reflect many of the broad patterns of the underlying data used for the analysis (Fig. 3). Generally, ecoregion boundaries most closely resemble depth, geomorphology and seabed temperature with productivity and ice cover tending to contain stronger within ecoregion variation (e.g. around polynyas or persistent productivity hotspots, Fig. 3). The Southern Ocean is dominated by deeper environments with over 85% of the seabed deeper than 2000 m (Fig. 3). This is also reflected in the geomorphology where over 75% of the Southern Ocean is made up of three geomorphic types, Rugose Ocean Floor, Abyssal Plain and Lower Slope (Fig. 3). The Atlantic basin, the largest ecoregion, is dominated by depths greater than 4500 m.

Within the deeper environments of the Southern Ocean, the primary drivers of the classification were the distribution of rare and mostly isolated shallow habitats often associated with seamounts, seamount ridges, plateaus and islands. Also important was the presence of mid-ocean ridges and large plateaus that separate deeper habitats. On the shelf and slope, where survey effort is higher, biological data on species distributions (in the form of previous biogeographical research) were the key drivers. Therefore, our results broadly reflect that of Spalding et al. [2], the Delegations of Australia and France, [17] and Clarke et al. [33] with some boundaries adjusted to more closely reflect the distribution of bathomes and geomorphic features.

In contrast to most other recent benthic classifications in the region examples include: [2], [17], [32], the results of this study encompass the deeper environments of the Southern Ocean as well as the continental shelf and islands. While the Global Oceans and Deep Seabed (GOODS) biogeographic classification provides a global scale classification, the only previous benthic classification to include these deeper environments at a circum-Antarctic scale was exploratory and incomplete [26]. This exploratory classification identified 20 regions using the statistical relationships between three physical datasets of depth, slope and seabed temperature. The classification presented here incorporates known or inferred relationships between the benthic biology and the physical environment and their influence on barriers to dispersal. The seamount classification provided by Clark et al. [120] provides a biologically meaningful classification of seamounts and we also consider two of the four criteria used (i.e. summit depth and seamount proximity). However, the Clark et al. [120] classification incorporates seamounts i.e. [121] identified using a technique we found to overestimate the number of seamounts and therefore preferred the hand drawn seamounts identified by O’Brien et al, [64]. For the continental shelf and islands, this work provides a physical underpinning and whole of Southern Ocean context for the patterns in biodiversity found by Clarke et al. [33], the Delegations of Australia and France, [17] and Spalding et al. [2]. In addition to the identification of the regions themselves, we also offer a classification of the seabed habitats contained within each ecoregion.

Environmental types were mostly identified by nesting together ecoregions, bathomes and geomorphic features to avoid the potential for false within-class homogeneity [115]. However, there were some exceptions. To add utility to MPA planning, we reflect the variety of three geomorphic features by classifying seamounts and seamount ridges by only their shallowest bathome and canyons by whether they commence on the shelf or slope. When an MPA is placed to protect the benthos of integral physical features (e.g. those likely to contain vulnerable marine ecosystems) that have a high level of connectivity among the habitats they contain, such as seamounts, seamount ridges or canyons, the feature should be included as a whole and therefore the internal bathome changes become less relevant to spatial MPA planning [122]. The coastal terrane of some islands and the Antarctic continent represent areas adjacent to rugged coastlines with highly irregular depth structuring which could not be fully resolved with the depth data used and were therefore not further classed by bathomes [64]. Future MPA planning within regions where finer-scale bathymetry is available should further class these environmental types by the bathomes they contain and could be guided by the framework we provide. A level 2 nesting (see Fig. 1) provides a broader spatial management unit. For instance, a level 2b nesting of geomorphic features within ecoregions identifies 210 types. However, caution should be used when not further nesting geomorphic features within bathomes (i.e. level 3 of Fig. 1) since this can result in false within-class homogeneity [115]. Some environmental types are described within the ‘restricted environmental types’ section below.

According to the advice of the Science Committee to CCAMLR, we treat the benthic and pelagic environments separately [26], [123]. The assumption is that these environments are decoupled due to the extreme depths and complex topography and oceanography of the Southern Ocean [75]. However, there is increasing evidence of interaction, and coupling may be favoured by intense seasonality and quick and efficient cycling through the pelagic food web [47], [75], [81], [91], [124]. We partially account for this interaction by considering sea surface productivity, the duration of ice cover and frontal systems within the analysis.

The classification we provide aims to capture the larger scale patterns by incorporating a combination of physical and biological data and also taking into consideration probable barriers to dispersal. As new data become available they can be used to refine the classification. Increasing knowledge, especially of cryptic species (i.e. species that are morphologically similar but differ genetically) seems most likely to lead to adjustment of the boundaries and the identification of further within-region heterogeneity, and not to a fundamentally different view of the distribution of habitats. Biogeographic classifications aim to identify general patterns within complex and diverse systems and therefore false heterogeneity for some species is inevitable. However, for the purposes of MPA planning, incorporating the general geographic and depth distribution patterns will increase the chance of representing the range of species habitat requirements within the final MPA system. The ecoregions and the environmental types can be used to consider the representativeness of biodiversity in a system of MPAs across the whole of the Southern Ocean.

### Assessment of Representativeness

**Figure 4:**
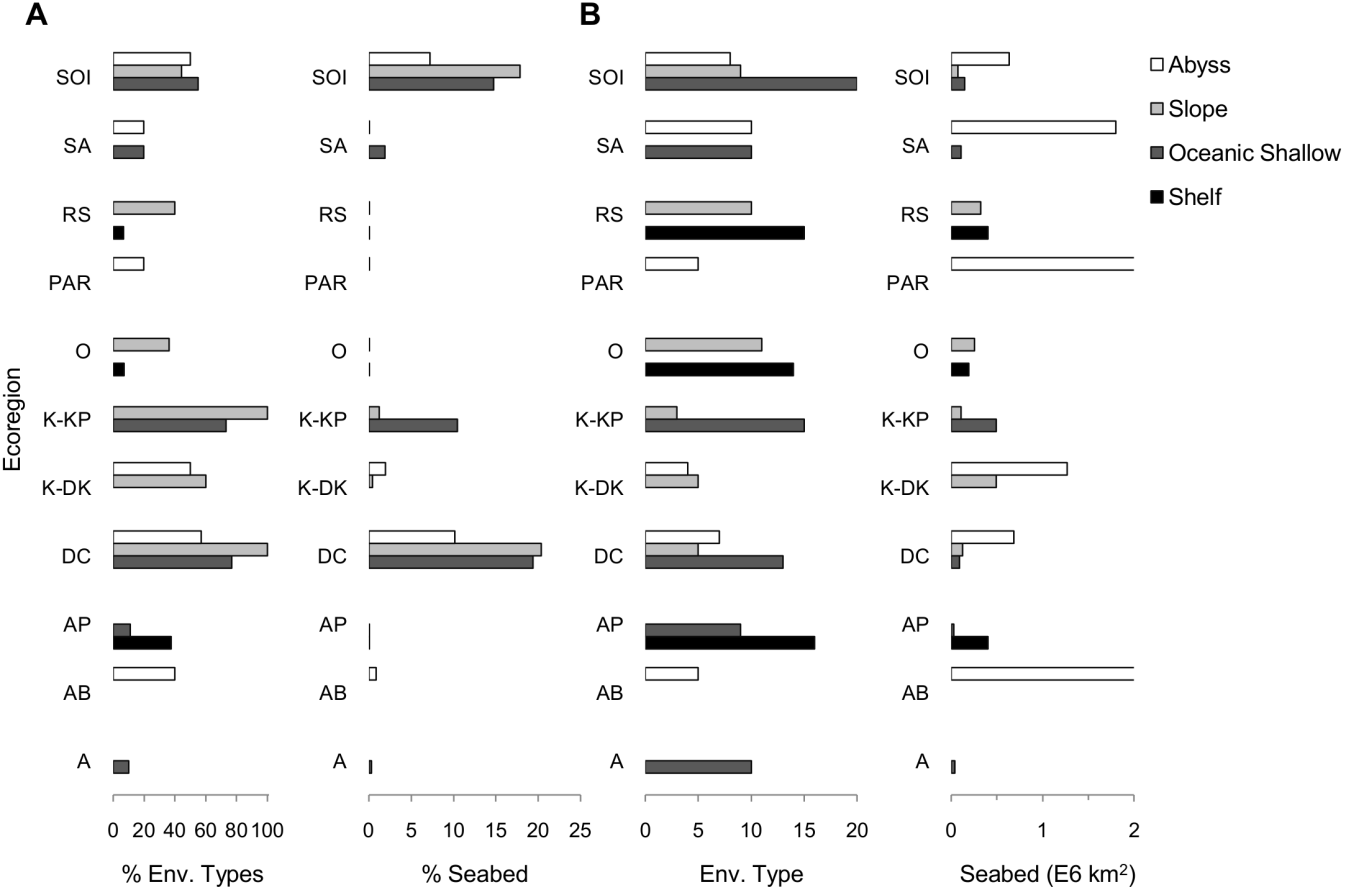
Marine protected areas and their spatial coverage of ecoregions and representation of environmental types. **A)** The proportion of the seafloor and the proportion of environmental types included either partly or wholly within existing MPAs are displayed within four broad geomorphic classes. These classes are further described in Table 3 and are represented within the plots from top to bottom as Abyss (white), Slope (light grey), Oceanic Shallow (dark grey) and Continental Shelf (black). **B)** The total number of environmental types and seafloor area for each geomorphic class are also shown. The incomplete bars within PAR and AB have values of 2.95 E6 km^2^ and 6.59 E6 km^2^ respectively. The ecoregion code names are: SOI (South Orkneys Islands), SA (South Atlantic), RS (Ross Sea), PAR (Pacific-Antarctic Ridge), O (Oates), K-KP (Kerguelen-Kerguelen Plateau Subregion), K-DK (Kerguelen-Deep Kerguelen Subregion), DC (Del Cano), AP (Antarctic Peninsula), AB (Atlantic Basin), A (Amundsen).

We found the level of existing representation for each environmental type across geomorphic classes is low (figure 4). None of the 23 ecoregions contains a system of MPAs that is representative of the benthic biodiversity they contain, with 12 ecoregions having no MPAs. Of the remaining 11 ecoregions, none includes a system of MPAs that sample each of the environmental types present in the ecoregion. The three ecoregions which perform the best are 1) Del Cano where South Africa declared the Prince Edward Islands MPA in 2012, 2) the South Orkney Islands, where CCAMLR established its first entirely high seas MPA in 2009 and 3) the Kerguelen Plateau where Australia and France have established MPAs. For some geomorphic classes, the number of environmental types in MPAs especially within the Ross Sea, Oates, Deep Kerguelen and Antarctic Peninsula ecoregions, is greater than 20% (figure 4). However, the area of seabed in MPAs for these geomorphic classes is less than 1% suggesting that only a small portion of each environmental type is represented in MPAs.

We have provided an assessment of progress toward a representative system of MPAs. The primary aim of the analysis described here is to identify important gaps in the protected area system, particularly related to ecoregions and environmental types. We have not identified how the biodiversity within a region needs to be adequately included in MPAs in order to achieve viability of the protected biodiversity; requirements will vary between different ecoregions and environmental types.

There are a number of existing conservation planning processes directed towards resolving some of these gaps in the existing system of MPAs (Fig. 2), including Eastern Antarctica [17] and the Ross Sea for example: [106], [125]. These proposals will help fill the gaps for achieving a Southern Ocean system of MPAs. Analyses such as the one we present here could be used to help assess progress towards a representative system of MPAs covering each of the ecoregions and which areas may be most suitable for filling gaps. An accounting procedure to help assess the adequacy of MPAs would improve future assessments.

#### Restricted environmental types

In this study we contribute to Southern Ocean MPA planning processes by identifying environmental types with a restricted spatial distribution and within ecoregions largely lacking any existing or proposed representation in MPAs. We identify 107 restricted environmental types (Fig. 2). The distribution of restricted environments is driven by rare environmental units that arise when a component of the hierarchical classification is scarce. The island arc, a geomorphic feature within the Southern Sandwich Islands ecoregion, is the only habitat of its kind in the Southern Ocean. Therefore the ecosystems associated with the particular environments created where a ridge of volcanic islands is formed adjacent to a subduction zone can only be protected in this location. Other restricted environments arise from an uncommon combination of geomorphic features and bathomes. There are many seamounts within the Pacific basin ecoregion however there are only two, the Belgica Guyot and the Lecointe Guyot, that have very shallow mounts of less than 200 m depth. Furthermore, there are only five discrete locations within the CCAMLR region where these shallow seamount environmental types occur.

Maud Rise is another example of a biologically important region containing restricted environmental types. At Maud Rise, oceanography, sediment characteristics and sea ice processes have been linked to high biodiversity throughout all trophic levels from pelagic predators to benthic species [30]. Maud Rise is an underwater plateau and slope region with two associated seamounts within the Atlantic Basin ecoregion. Four of the six environmental types of Maud Rise are restricted. These include: 1) the only seamount in the ecoregion with a mount in the –1500 to –2000 m bathome; 2) the only seamount ridge within the ecoregion with a mount in the –1000 to –1500 m bathome and; 3) the only plateau in the ecoregion with two environmental types resulting from plateau areas within the –2000 to –3000 m bathome and –3000 to –4500 m bathome. The Maud Rise seamount is suspected to contribute to the most recurring open-ocean polynya in the Southern Ocean which forms over Maud Rise [30], [126]. These polynyas drive convection which influences interaction between surface productivity, the benthos and the upwelling of nutrients all of which may contribute to the rich and prospering food web in the region [30].

### Conclusion

The international effort overseen by CCAMLR to establish a representative network of MPAs within the Southern Ocean has already made a significant step in declaring the world’s first MPA within the high seas located outside national jurisdiction. The efforts to achieve the conservation commitments made by CCAMLR and parties to the Antarctic Treaty sets a precedent for conservation within other high seas regions of the global ocean [101]. However the current levels of representation clearly contain significant gaps that future conservation planning efforts need to address. The environmental types presented here are an improvement on previous statistical classifications because, rather than opting for a method that identifies statistically dissimilar areas within the data, we have identified environmental types using known and probable relationships between biogeographic patterns, environmental drivers, and knowledge of dispersal of benthic taxa. The classification of environmental types allows the gaps in the MPA network to be quantified. It also provides a new circum-Antarctic map of environmental types that can be used to support spatial management aimed at conserving benthic biodiversity across the entire Southern Ocean.

## Supporting Information

Table S1 **Spatial coverage of ecoregions, geomorphic features and environmental types within the Southern Ocean.**
(XLSX)

Text S1 **Detailed description of ecoregion boundaries.**
(DOC)

Text S2 **Outline of methods used to identify geomorphic features.**
(DOCX)
