# Supplementary material for: A Hierarchical Classification of Benthic Biodiversity and Assessment of Protected Areas in the Southern Ocean
Source: PLoS One. 2014 Jul 17;9(7):e100551. doi: 10.1371/journal.pone.0100551 (PMC4102490; doi:10.1371/journal.pone.0100551)
Supplement: Text S1 — Detailed description of ecoregion boundaries. (DOC) [file pone.0100551.s002.doc]

**Supporting Information**

**Text S1: Detailed description of ecoregion boundaries**

A hierarchical classification of benthic biodiversity

and assessment of protected areas in the Southern Ocean

L.L. Douglass*, J. Turner, H. S. Grantham, S. Kaiser, A. Constable, R. Nicoll,

B. Raymond, A. Post, A. Brandt, D. Beaver

^*^Corresponding author: [douglass.lucinda@gmail.com](mailto:douglass.lucinda@gmail.com)

The ecoregions of the Southern Ocean were delineated as per the methods described in the main manuscript. Here we provide further discussion of each boundary definition. Unless otherwise stated, the conclusions discussed below are drawn from the authors observations of the data used within the analysis.

We initially separated regions containing habitats more likely to be associated with the shallower shelf and slope of the Antarctic continent and those associated with the deeper ocean using the 4000 m bathymetric contour. We deemed the 4000 m, rather than the previously defined 3000 m, bathymetric contour a more appropriate boundary . This was especially true for ecoregions based on oceanic ridges and ocean basins. For example, two locations that have many seamounts and ridges that extend beyond 3000 m are; 1) Astrid Ridge at the western end of the Dronning Maud Ecoregion and; 2) the rift zone associated with the mid-ocean ridge between the Antarctic and African plates in the Atlantic sector. Therefore, a 3000 m boundary would not adequately distinguish between these shallower and deeper ecological systems.

The 4000 m boundary was used except for parts of the East Indian Abyssal, Kerguelen, Pacific Basin Ecoregions and Pacific-Antarctic Ridge. These regions were mapped to better capture geomorphology. Where the structural slope of the Oates Ecoregion extends past the 4000 m contour in the Indian Ocean sector, the boundary of the structural slope was used in preference to the 4000 m contour. The boundary between the Kerguelen Ecoregion and the Antarctic continent was defined by the southern limit of the mapped contourite drift to the east of the Kerguelen Plateau, the mapped base of the slope of the broader Kerguelen Plateau (i.e including Banzare and Elan Banks) and the canyon that intersects with the broader Kerguelen Plateau slope at 77.5^o^E, 61.5^o^S. Similarly to Spalding et al. , we included the seamounts and the seamount chain near 120^o^W within the Amundsen Ecoregion of the Antarctic continent. The boundary between the Pacific-Antarctic Ridge Ecoregion and the Antarctic continent was defined by the continental limit of the rugose ocean floor, a line from 170.5^o^E, 70.5^o^S to 171.2^o^E, 69.1^o^S, the 3000 m bathymetric contour, a line from 177.4^o^E to 178.8^o^E at 70.6^o^S to include Scott Canyon in its entirety, and by the northern limit of the Ross Sea Ecoregion slope and continental rise.

# Boundaries between Deeper Ocean Ecoregions

### Atlantic Basin – South Georgia

The shallower regions of the North Weddell Ridge forms a barrier to water circulation and potentially dispersal for species between the abyssal habitats of the Atlantic Basin and the South Georgia Ecoregions . We delineate this distinction using the 4000 m bathymetric contour. However, there may be movement from the Weddell abyss to the Atlantic Ocean through the large South Sandwich Trench and between the South Orkney and Bruce plateaus . The Atlantic Basin Ecoregion is characterized by the colder, very deep seafloor associated with rugose ocean floor and the Weddell Abyssal Plain. At more than 1000 km apart the abyssal plains of the Weddell Sea and South Georgia Basin Ecoregions are likely to be isolated. The seamounts and seamount ridges associated with North Weddell Ridge are also distant (depths >250 km) from those associated with the North Scotia Ridge, Falkland Ridge and the Islas Orcadas Rise.

### South Atlantic – South Georgia:

The 4000 m contour is used to divide the shallower habitats (depths <3000 m) of the Mid-Atlantic Ridge from habitats at similar depths within the South Georgia Ecoregion which are more than 600 km to the west. This distance is likely to form a barrier to dispersal for some benthic species. The South Atlantic Ecoregion is also characterized by being shallower and less productive than the adjacent areas of deep sea within the South Georgia Ecoregion.

### Atlantic Basin – South Atlantic:

The abyssal habitats of the Atlantic Basin Ecoregion and the South Atlantic are separated by the Mid Atlantic and Southwest Indian Ridges, defined here by the 4000 m bathymetric contour, which influences ocean circulation and is a potential barrier to dispersal . The eastern border is defined by a combination of the 4000 m contour and the eastern edge of the Andrew Bain Trough at approximately 53°S, 28°E.

### South Atlantic – Del Cano:

The 4000 m contour and the boundary between CCAMLR subareas 58 and 48 (Atlantic and Indian Ocean sections of the Southern Ocean) is retained as the boundary between the South Atlantic and Del Cano Ecoregions. This represents a reasonable midpoint between the shallower habitats (depths <2000 m) of the South Atlantic Ecoregion which are more than 200 km from habitats at similar depths within the Del Cano Ecoregion.

### Atlantic Basin – Del Cano:

The 4000 m contour is used to divide the shallower habitats (depths <2000 m) associated with the Southwest Indian Ridge, Del Cano Rise and the Crozet and Prince Edward Islands from the deeper waters of the Atlantic Basin Ecoregion. There are no habitats shallower than 3000 m within the Indian Ocean sector of the Atlantic Basin Ecoregion.

### Atlantic Basin – Ob & Lena:

A 100 km buffer on the southern limit of the plateau slopes and seamount ridges of the Ob & Lena Ecoregion separates these shallow habitats from the deeper waters of the Atlantic Basin Ecoregion. The northern limit of the mapped abyssal plain within the Atlantic Basin Ecoregion is used in preference to the buffer for the eastern section of the boundary.

### Del Cano – Ob & Lena:

The boundary between the Ob & Lena and Del Cano Ecoregions is described by a combination of a 100 km buffer on the northern limit of the plateau slopes of the Ob & Lena Ecoregion, the boundary between CCAMLR subareas 58.4 and 58.6, and the southern limit of the abyssal plain mapped within the Del Cano Ecoregion. This represents a reasonable boundary between the warmer Del Cano Ecoregion (which is to the north of the Polar Front ) and the colder Ob & Lena Ecoregion. The shallower habitats at depths <2000 of the system of plateaus, seamounts and seamount ridges that lie between 40^o^E, 50^o^S and 60^o^E, 60^o^S within the Ob & Lena Ecoregion are separated by more than 300 km from the habitats at similar depths within the Del Cano Province. This distance and the difference in seafloor temperature may represent a barrier to dispersal for many benthic species. The polar front will also contribute as a barrier especially for species that rely on epipelagic larvae to provide connectivity between distant populations .

### Atlantic Basin – Kerguelen:

The northern limit of the Valdivia Abyssal Plain was used as the boundary between the Atlantic Basin and Kerguelen Ecoregions. This boundary separates the seamounts and seamount ridges more likely to be associated with the broader Kerguelen Plateau (including Elan and Banzare Banks) from those that are more likely to be part of the Valdivia abyssal system. The seamounts and seamount ridges of the Kerguelen Ecoregion are relatively distant (depths >200 km) from the similar habitats mapped within the Atlantic Basin Ecoregion.

### Ob & Lena – Kerguelen:

The boundary between the Ob & Lena Ecoregion and the Kerguelen Ecoregion is described by a combination of a 100 km buffer on the seamounts and seamount ridges of the Ob & Lena Province, the eastern limit of the ocean trough mapped at 60^o^E, and a line from 61.3^o^E, 51.5^o^S to 55.2^o^E, 49.4^o^S. This boundary is intended to separate the ridges, seamounts and seamount ridges that are more likely to be associated with the Kerguelen Plateau from those that are more likely to be a part of the Ob & Lena Ecoregion. The seamounts and seamount ridges of the Kerguelen Ecoregion are all separated from those within the Ob & Lena Ecoregion by more than 200km. The northern boundary of the Ob & Lena Ecoregion describes the approximate boundary between the warmer sea floor of the Kerguelen Ecoregion and the colder waters of the Ob & Lena Ecoregion. The Ob & Lena and the Kerguelen Ecoregions are characterized by the presence of shallower habitats (depths<3000 m) surrounded by deeper ocean which forms a barrier separating the shallow habitats by more than 400km.

### Del Cano – Kerguelen:

The boundary between Del Cano and Kerguelen Ecoregions are formed by the eastern limit of the abyssal plain mapped within the Del Cano Ecoregion. The Del Cano and Kerguelen Ecoregions are characterized by the presence of shallower habitats (depths <3000 m) surrounded by deeper ocean separating the shallower habitats by more than 650km.

### Kerguelen Sub-regions:

Three sub-regions are described within the Kerguelen Ecoregion: 1) Deep Kerguelen; 2) Kerguelen Plateau and; 3) Banzare Bank. The Kerguelen Plateau and Banzare Bank sub-regions are defined by the enclosing 2500 m bathymetric contours while the Deep Kerguelen sub-province contains all remaining areas within the Kerguelen Province including Elan Bank. The shallower habitats occurring within the Kerguelen Plateau Sub-province (depths <1500m), the Banzare Bank Sub-province (depths of 500 to1500 m) and the Deep Kerguelen Sub-province (depths of 1000 to 1500 m) are distant from each other by more than 200km. This distance in conjunction with the movement of frontal waters through the Fawn Trough may constitute a barrier to dispersal for some benthic species. The Kerguelen Plateau and Banzare Bank Sub-provinces contain no deep ocean habitats (depths >3000m).

### Kerguelen – East Indian Abyssal

The 4000 m contour describes the boundary between the Kerguelen and East Indian Abyssal Provinces. The abyssal habitats of the west Indian Ocean sector of the CCAMLR region and the Kerguelen Ecoregions may be connected to those within the Atlantic Basin Ecoregion through the Fawn or Princess Elizabeth Troughs. However, these abyssal habitats are more likely to be biologically isolated since they are separated by over 600 km at similar depths. Also, dispersal between the Antarctic continent and Kerguelen Plateau will be influenced by regional currents. For instance, westward dispersal will be hindered by a mostly eastward flowing current except for a westward current along the slope . Two cyclonic gyres exist either side of 80°E . When compared to the other two ecoregions dominated by depths greater than 4500 m namely the Atlantic Basin and Pacific Basin ecoregions, the East Indian Abyssal is the shallowest. The East Indian Abyssal ecoregion is closer in sea bed temperature to the Indian Ocean section of the Atlantic Basin that the other ecoregions dominated by very deep ocean (depths >4500m). All the very deep ocean ecoregions have consistently low sea surface productivity.

### East Indian Abyssal – Pacific-Antarctic Ridge:

The boundary between the East Indian Abyssal and Pacific-Antarctic Ridge Ecoregions is described by a line from 147.9^o^E, 62.1^o^S to 136.9^o^E, 55^o^S. This describes the approximate boundary between the colder deeper waters of the East Indian Abyssal Ecoregion and the shallower, warmer waters of the Pacific-Antarctic Ridge. The line also separates the isolated (by >200 km) seamount ridges of the East Indian Abyssal Ecoregion from similar habitats within the Pacific-Antarctic Ridge Ecoregion. The Pacific-Antarctic Ridge Ecoregion is characterized by presence of large extents of shallower habitats (depths of <2000 m) which are virtually absent from the East Indian Abyssal Ecoregion.

### Pacific-Antarctic Ridge - Pacific Basin

The boundary between colder deeper waters of the Pacific-Antarctic Ridge and the warmer shallower waters of the Pacific Basin Ecoregions are described by a line from 171.9^o^W, 70.1^o^S to 140.6^o^W, 60^o^S. The abyssal habitats that characterize the Pacific Basin Ecoregion are predominantly absent from the Pacific-Antarctic Ridge Ecoregion. Also, the abyssal depths of the Pacific Basin Ecoregion are isolated from other deep ocean ecoregions (ie. Pacific-Antarctic Ridge, East Indian Abyssal, South Georgia and Weddell Sea Ecoregions) due to a distance of more than 200 km between habitats at similar depths and the variation of seafloor temperature.

# Boundaries among Ecoregions of the continental shelf and slope

In most cases the boundaries between the continental ecoregions are as described by Spalding et al. , Constable et al. or Clarke et al. . In each case, these boundaries were adjusted to reflect the distribution of bathomes and geomorphic features. For example, canyons were used as boundaries where these coincided with the approximate location of previously proposed boundaries since canyons may hinder the dispersal of some benthic species.

### Antarctic Peninsula – Weddell Shelf:

The boundary proposed by Spalding et al. is adjusted slightly to reflect the mapped geomorphology and to extend the boundary into deeper waters. The boundary separates the colder water masses of the west Weddell Sea from the warmer waters of the western Antarctic Peninsula. This change in water temperature and the dramatic change in sea ice conditions represent an abrupt cline in the physical environment. The very high levels of sea ice that extend from the Larsen to the Ronne-Filchner ice shelves and the much colder sea floor temperatures over the continental shelf and slope represent a potential barrier to dispersal for species between East and West Antarctica. Spalding et al. also propose an ecoregion for the South Shetland Islands. The South Shetland Islands are on the Drake Plate with an active spreading ridge between the Drake and Antarctic Plates causing volcanic activity and unique ecological conditions that may act as a barrier to dispersal. However, low levels of endemism have been found with 5% of Molluscs studied by Linse et al. , and less than 10% endemism across all classes studied by Griffiths et al. . Therefore, the South Shetland Islands province proposed by Spalding et al. was included within the Antarctic Peninsula Ecoregion. Levels of endemism double when these ecoregions are considered together . The Weddell Sea region with approximately 20% endemism for Molluscan taxa, has higher levels of endemism than the adjacent Antarctic Peninsula Ecoregion .

### Antarctic Peninsula - South Orkneys:

The boundary of CCAMLR subarea 48.2 is retained as the boundary between the Antarctic Peninsula and the South Orkneys. However, the boundary was slightly adjusted to retain the mapped abyssal plain within the South Orkney Ecoregion. The South Orkney Islands are the exposed part of a continental fragment. The South Orkney Ecoregion is characterized by a colder sea floor than the Antarctic Peninsula. Also, the shallow shelf environments at depths of <200 m are distant (>200 km) from those on the Antarctic Peninsula. Linse et al. found molluscan endemism within the South Orkney Islands of 20%. Griffiths et al. found gastropod endemism of 25% and either low or no endemism in the other classes studied.

### South Orkneys – South Georgia:

The boundary between the South Orkneys and South Georgia Ecoregions is defined by the fracture zone that extends from the CCAMLR boundary at 50^o^W, 56.7^o^S to the bottom of the North Scotia Ridge at 43.7^o^W, 54.6^o^S and by a 100 km buffer on the 1500 m contour from the eastern end of the fracture zone to the South Sandwich Trench. The South Georgia Ecoregion contains a number of shallower habitats (depths < 1500 m) associated with the South Georgia Islands, the North Scotia Ridge, Ewing Bank and seamounts and seamount ridges within the South Georgia Ecoregion. These habitats are unlikely to be connected to similar habitats elsewhere. The shallower habitats around the South Georgia Islands are more likely to have connections with similar habitats around the Falkland (Malvinas) Islands to the west via the North Scotia Ridge than to those associated with the South Sandwich Islands, Bruce Ridge or the South Orkney Islands due to relative isolation (distance of similar habitats >200 km) and different frontal systems . The South Georgia Ecoregion has high persistent sea surface productivity probably caused by the upwelling of nutrients caused by perturbation of the southern polar front by the topology of the South Georgia Islands . Within the South Georgia Islands, Linse et al. found molluscan endemism to be 33% and Griffiths et al. found gastropod endemism to be 36%, bivalve endemism to be 13%, cheilostome endemism to be 15% and cyclostome endemism to be 5%.

### South Orkneys – South Sandwich:

The boundary between the South Orkneys and South Sandwich Ecoregions is a line from 32.7^o^W, 55.4^o^S to 30.0^o^W, 60.0^o^S, in addition to a curved line to include the seamount ridges at 30^o^W within the South Sandwich Ecoregion. This includes the higher productivity areas associated with the South Sandwich Islands and the deeper waters associated with the South Sandwich Trench. The active spreading ridge between the Sandwich and Scotia Plate occurs at around 30^o^W and this may form a barrier to dispersal to some benthic species . The shallower habitats (of depths <500 m) of the South Sandwich Islands are distant, by more than 350km, from habitats at similar depths within the South Orkneys Ecoregion. Linse et al. found two endemic species of Molluscs within the South Sandwich Islands. Griffiths et al. found gastropod endemism of 27% with low levels of endemism or no endemism within the other classes studied.

### South Sandwich – South Georgia:

The boundary between the South Sandwich and South Georgia Ecoregions is formed by the South Sandwich Trench. The South Sandwich Trench, formed by the subduction of the South American plate , is the deepest part of the CCAMLR region and is likely to form a barrier to dispersal for many benthic species. The shallower habitats (depths <1500 m) of the South Sandwich Islands are also distant (>350 km) from habitats at similar depths within the South Georgia Ecoregion.

### Weddell Shelf – Dronning Maud:

Both Spalding et al. (2007) and Clarke et al. (2007) propose 0^o^ as the boundary between the Weddell Shelf and Dronning Maud Ecoregions. This is the location of the Trolltunga Ice Tongue which may act as a barrier to dispersal between the two ecoregions. Trolltunga is a large ice tongue fed by the Jutulstraumen glacier which extends 40 km past the shelf break into the deep ocean between the 3000 and 4000 m bathymetric contours . Linse et al. (2006) found endemism of 19% among Molluscs in the Dronning Maud Ecoregion. Griffiths et al. (2009) found endemism of 21% for gastropods and 0% for the other three classes studied.

### Dronning Maud – Central Indian:

The Dronning Maud and Central Indian Ecoregions are separated by a line at 55°E. This boundary was proposed by Constable et al., since it is where wind and sea ice movement vectors diverge influencing oceanography and potentially dispersal. The Enderby Ecoregion proposed by Spalding et al. has been included within the Dronning Maud Ecoregion as implied by Constable et al. .. The Enderby Ecoregion is not as supported by recent biogeographic studies with Linse et al. , finding low levels of endemics in this area (5%).

### Central Indian Sub-regions:

The boundaries between the Central Indian sub-regions are as per Constable et al., who propose four sub-regions within the Central Indian Ecoregion; 1) West Kerguelen; 2) Prydz Bay; 3) East Kerguelen and; 4) Wilkes. Prydz Bay is defined by the influence of the Prydz Bay Gyre, while the boundary between the East Kerguelen and Wilkes sub-regions is based on the complex oceanographic conditions at about 110^o^E which may form a barrier to dispersal for some species .

### Central Indian - Oates:

The boundary between the Central Indian and Oates Ecoregions is as per Constable et al. who proposed 138^o^E as a boundary due to the divergence of wind and sea ice movement vectors which may represent a barrier to dispersal for some species.

### Oates – Ross Sea

Cape Adare at around 170^o^E is the boundary proposed by Spalding et al. , Clarke et al. and Ainley et al. for the Ross Sea. The Ross Sea is characterized by containing a shelf persistently high in productivity with a colder seafloor, Antarctica’s largest ice shelf cavity and habitat types which are distant (>200 km) from those found in adjoining ecoregions. For example, the numerous canyons incising the Antarctic slope in the Ross Sea Ecoregion are more than 1000 km from the Indian Ocean canyons and more than 300 km from the nearest canyons within the Amundsen Ecoregions. Linse et al. found endemism of 12% within the molluscan fauna of the Ross Sea. Griffiths et al. found endemism of 16% in gastropods and less than 10% in the other classes studied.

### Ross Sea – Amundsen

The boundary proposed by Clark et al. and Spalding et al. at 145^o^W was moved to approximately 150^o^W. The previous boundary was a default boundary for the region for which there was no data . The boundary we use at approximately 150°W is delineated by the last of the Ross Sea canyons on the slope and the beginning of the extensive cross shelf banks to the west of Stephen Island on the shelf. These shelf banks are almost 100 km in width and at less than 100 m in depth represent the largest single extent of coastal waters on the Antarctic shelf not permanently covered by ice. These extensive shallow banks may form a barrier to dispersal to deeper shelf species. This location is also the approximate location where wind vectors diverge from northerly winds at the western edge of the Amundsen Ecoregion, to the southerly winds in the adjacent area of the Ross Sea Ecoregion .

### Amundsen – Antarctic Peninsula

Clark et al. proposed 80^o^W as the boundary of the Western Antarctic Peninsula. This is an approximate boundary between the warmer, low sea ice waters of the Western Antarctic Peninsula and the highly productive, high sea ice conditions of the Amundsen Ecoregion. Biological sampling within the Amundsen Ecoregion is very low . However, the Admundsen Ecoregion contains a distinctive physical environment high levels of sea ice and geomorphic features (ie. canyons, cross shelf valleys, seamounts and seamount ridges) that are distant (>200 km) from those in adjacent regions. Also, the Amundsen ecoregion, Central Indian-Prydz Bay, Ross Sea and Antarctic Peninsula ecoregions have high productivity relative to the other ecoregions associated with the Antarctic continent.

**References**

1. Clarke A, Johnston N (2003) Antarctic marine benthic diversity. Oceanography and Marine Biology, an Annual Review, Volume 41: An Annual Review 41: 47-114.

2. Spalding M, Fox H, Allen G, Davidson N, Ferdana Z, et al. (2007) Marine ecoregions of the world: a bioregionalization of coastal and shelf areas. Bioscience 57: 573-583.

3. Robertson R, Visbeck M, Gordon A, Fahrbach E (2002) Long-term temperature trends in the deep waters of the Weddell Sea. Deep Sea Research Part II: Topical Studies in Oceanography 49: 4791-4806.

4. Sokolov S, Rintoul SR (2009) The circumpolar structure and distribution of the Antarctic Circumpolar Current fronts. Part 1: Mean circumpolar paths. Journal of Geophysical Research - Oceans 114: 1-19.

5. Marques A, Peña Cantero A (2010) Areas of endemism in the Antarctic–a case study of the benthic hydrozoan genus Oswaldella (Cnidaria, Kirchenpaueriidae). Journal of Biogeography 37: 617-623.

6. Heywood K, Sparrow M, Brown J, Dickson R (1999) Frontal structure and Antarctic bottom water flow through the Princess Elizabeth Trough, Antarctica. Deep Sea Research Part I: Oceanographic Research Papers 46: 1181-1200.

7. Meijers AJS, Klocker A, Bindoff NL, Williams GD, Marsland SJ (2010) The circulation and water masses of the Antarctic shelf and continental slope between 30 and 80^o^E. Deep Sea Research II 57: 723-737.

8. Nicol S, Pauly T, Bindoff N, Wright S, Thiele D, et al. (2000) Ocean circulation off east Antarctica affects ecosystem structure and sea-ice extent. Nature 406: 504-507.

9. Constable AJ, Raymond B, Doust S, Welsford D, Martin-Smith K (2010) Elaborating a representative systems of marine protected areas in eastern Antarctica, south of 60oS. Report to the Commission on the Conservation of Antarctic Marine Living Resources (CCAMLR) XXIX, Working Group on Ecosystem Monitoring and Management. Document number: WG-EMM-10/26.

10. Clarke A, Griffiths H, Linse K, Barnes D, Crame J (2007) How well do we know the Antarctic marine fauna? A preliminary study of macroecological and biogeographical patterns in Southern Ocean gastropod and bivalve molluscs. Diversity and Distributions 13: 620-632.

11. Linse K, Griffiths H, Barnes D, Clarke A (2006) Biodiversity and biogeography of Antarctic and sub-Antarctic mollusca. Deep Sea Research Part II: Topical Studies in Oceanography 53: 985-1008.

12. Griffiths H, Barnes D, Linse K (2009) Towards a generalized biogeography of the Southern Ocean benthos. Journal of Biogeography 36: 162-177.

13. Griffiths H, Linse K, Barnes D (2008) Distribution of macrobenthic taxa across the Scotia Arc, Southern Ocean. Antarctic Science 20: 213-226.

14. Sokolov S, Rintoul SR (2009) The circumpolar structure and distribution of the Antarctic Circumpolar Current fronts. Part 2: Variability and relationship to sea surface height. Journal of Geophysical Research - Oceans 114.

15. Sokolov S, Rintoul S (2007) On the relationship between fronts of the Antarctic Circumpolar Current and surface chlorophyll concentrations in the Southern Ocean. Journal of Geophysical Research 112: C07030.

16. Trouw R, Passchier C, Simoes L, Andreis R, Valeriano C (1997) Mesozoic tectonic evolution of the South Orkney Microcontinent, Scotia arc, Antarctica. Geological Magazine 134: 383-401.

17. Barker PF (2001) Scotia Sea regional tectonic evolution: implications for mantle flow and palaeocirculation. Earth-Science Reviews 55: 1-39.

18. Walkden GJ, Heywood KJ, Nicholls KW, Abrahamsen P (2009) Freshwater transport at Fimbulisen, Antarctica. Journal of Geophysical Research 114: C08014.

19. Ainley DG, Ballard G, Weller J (2010) Ross Sea Bioregionalisation Part I: Validation of the 2007 CCAMLR Bioregionalization Workshop Results Towards Including the Ross Sea in a Representative Network of Marine Protected Areas in the Southern Ocean. CAMLR reference number: CCAMLR WG-EMM-10/11.

20. Griffiths H (2010) Antarctic Marine Biodiversity-What Do We Know About the Distribution of Life in the Southern Ocean? PLoS ONE 5: e11683.
