# Supplementary material for: A Hierarchical Classification of Benthic Biodiversity and Assessment of Protected Areas in the Southern Ocean
Source: PLoS One. 2014 Jul 17;9(7):e100551. doi: 10.1371/journal.pone.0100551 (PMC4102490; doi:10.1371/journal.pone.0100551)
Supplement: Text S2 — Outline of methods used to identify geomorphic features. (DOCX) [file pone.0100551.s003.docx]

**Supporting Information**

**Text S3: Outline of methods used to identify geomorphic features**

A hierarchical classification of benthic biodiversity

and assessment of protected areas in the Southern Ocean

L.L. Douglass*, J. Turner, H. S. Grantham, S. Kaiser, A. Constable, R. Nicoll,

B. Raymond, A. Post, A. Brandt, D. Beaver

^*^Corresponding author: [douglass.lucinda@gmail.com](mailto:douglass.lucinda@gmail.com)

The geomorphic features of the Southern Ocean as described by O'Brien et al. (2009), were mapped using GIS based on interpretation of bathymetry data. The key datasets used were the GEBCO08 bathymetry contours, which are derived from ship track data, and the ETOPO2 satellite bathymetry (Smith and Sandwell, 1997). Based on interpretation of the seafloor bathymetry, 28 geomorphic units were identified at a scale of about 1: 1-2 million. The geomorphic features were digitised in ArcGIS by hand as polygons based on the criteria shown in Table 3 of the manuscript (see O’Brien et al., 2009). In this classification, the International Hydrographic Organisation classification of undersea features (International Hydrographic Organisation, 2001) was used as a starting point which was expanded to accommodate additional features of the region and to recognise those that are likely to have differing substrates and influence on oceanography. This approach was used in order to improve the technique as a predictor of physical conditions that may influence benthic communities. Traditional classifications for continental slope and rise were not applied because distinguishing between slope and rise around much of Antarctica is very difficult due to the very gradual slope changes associated with the large sediment wedges supplied to the margin by glaciation.

The ETOPO2 data comprises a bathymetric grid derived by inverting satellite gravity measurements controlled by available ship based bathymetry. This grid provides a depth grid in areas where ship-based data are not available, of particular importance in the Southern Ocean region where ship tracks can be sparse. The limitations of ETOPO2 for this study are that the method has problems resolving features less than 12.5 km across and there are uncertainties in the absolute accuracy of depth values, particularly for areas of continental shelf (Marks and Smith, 2006). To overcome some of these uncertainties, particularly for areas of shelf and slope, GEBCO contours were overlain on ETOPO2 to provide a cross check on the location and depth of features.

The classification was also calibrated against seismic lines sourced from the SCAR Seismic Data Library System. Seamounts and other rocky features protruding through the sediment cover were compared with the mapped extent of features based on the ETOPO2 data, and features were updated as necessary. In areas with detailed multibeam bathymetry, such as 50 m grid cell data obtained by the OGS Explora in 2006 from the continental slope off George V Land (De Santis et al., 2007), the geometry of canyons and slope ridges were compared to that obtained from the global bathymetry as a test of the global compilations. These comparisons showed good consistency in the position of features between the multibeam and global bathymetric datasets, though the global data lacks the detailed dimensions provided by the high resolution multibeam data.

The geomorphology boundaries do not perfectly align to the bathymetry for a number of reasons. Firstly, they were hand drawn which results in a smoother boundary than the pixels generated by the satellite bathymetry. Secondly, features are not defined by fixed depth intervals.  For instance, a boundary around a plateau or at the slope edge is defined based on the maximum change in slope, rather than a specific depth. Thirdly, we also used the Gebco bathymetry to define features where appropriate (e.g. in areas with enough shiptracks to provide good data coverage).

**References:**

De Santis, L., Brancolini, G., Accettella, A., Cova, A., Caburlotto, A., Donda, F., Pelos, C., Zgur, F., & Presti, M. 2007. New Insights into Submarine Geomorphology and Depositional Processes along the George V Land Continental Slope and Upper Rise (East Antarctica).In Cooper, A.K., & Raymond, C.R. eds. *Keystone in a Changing World - Online Proceedings of the 10th International Symposium on Antarctic Earth Sciences*. USGS Open-File Report 2007-1047, Extended Abstract 061, 5 pp.

International Hydrographic Organisation (2001) *Standardization of Undersea Feature Names: Guidelines Proposal Form Terminology* International Hydrographic Organisation and International Oceanographic Commission, Monaco.

Marks, K.M. and Smith, W.H.F., 2006.An evaluation of publically available global bathymetric grids. Marine Geophysical Researches, 27, 19-34.

O’Brien, P.E., Post, A.L., & Romeyn, R. 2009. Antarctic-wide geomorphology as an aid to habitat mapping and locating Vulnerable Marine Ecosystems.*In Commission for the Conservation of Antarctic Marine Living Resources Vulnerable Marine Ecosystems Workshop, Paper WS-VME-09/10.*La Jolla, California, USA: CCAMLR. Available online: data.aad.gov.au with search ID 'ant_seafloor_geomorph' as at November 2013.

Smith, W.H.F., & Sandwell, D.T. 1997. Global sea floor topography from satellite altimetry and ship depth soundings.*Science,* 277, 1956-1962.
